# Association between hepatitis B virus infection and colorectal liver metastasis: a meta-analysis

**DOI:** 10.1080/21655979.2021.1890871

**Published:** 2021-02-25

**Authors:** Rongqiang Liu, Weihao Kong, Mingbin Deng, Guozhen Lin, Tianxing Dai, Linsen Ye

**Affiliations:** a Department of Hepatic Surgery and Liver Transplantation Center, The Third Affiliated Hospital of Sun Yat-sen University, Guangzhou, China; b Department of Hepatobiliary Surgery, The First Affiliated Hospital of Guangzhou Medical University, Guangzhou, Guangdong, China; c Department of Emergency Surgery, The First Affiliated Hospital of Anhui Medical University, Hefei, Anhui, China

**Keywords:** Hepatitis B Virus, colorectal liver metastasis, meta-analysis

## Abstract

The paper aims to assess the association between Hepatitis B Virus infection and colorectal liver metastasis by conducting a meta-analysis. The relevant studies were searched until 24 July 2020, Studies that assessed the correlation between HBV infection and CRLM were recruited. A random effects model was applied to calculate the odds ratio (OR) with 95% confidence interval (CI). All data analyses were performed by STATA 12.0 software. Ten studies involving 17529 participants were included in the study. The results shown that there was obvious association between HBV infection and CRLM (OR: 0.51, 95% CI: 0.28–0.91). The study type and case–control rate may be the main causes of heterogeneity. In addition, HBV infection had no association with extrahepatic metastasis or prognosis of patients with CRLM. Sensitivity analyses confirmed that the results were stable, and Egg’s test indicated that there was no publication bias. Patients with HBV infection have the reduced risk of CRLM.

## GRAPHICAL ABSTRACT

**Figure:**
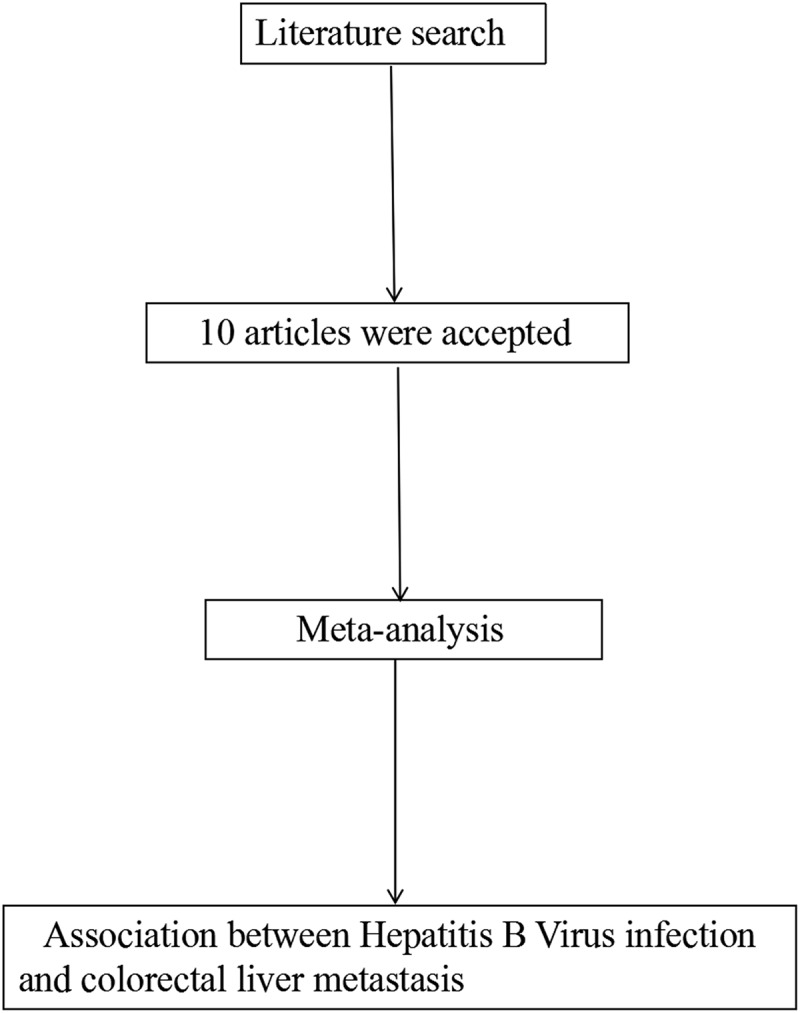

## Introduction

Colorectal cancer (CRC) is one of the most common malignant tumors, with the third highest incidence rate and the second highest mortality rate worldwide [1]. It is estimated that more than 1.9 million new cases of CRC and 0.94 million deaths occurred in 2020 [1]. The incidence of CRC has significantly increased in developing countries [2]. In China alone, the number of new colorectal cancer patients reached 388,000, and the number of deaths from colon cancer was as high as 187,000 in 2015 [3]. The liver is the most common location of colorectal cancer metastasis. The occurrence of liver metastases in patients with advanced colorectal cancer is about 35% to 40% [4]. It is estimated that more than half of CRC patients have liver metastases at autopsy [5]. Approximately 14% to 25% of CRC patients present with simultaneous colorectal liver metastases at the time of tumor diagnosis [6]. Even after the resection of the primary tumor, many patients would still develop metachronous liver metastases and die from liver metastases. CRLM is a key factor affecting the therapeutic effect and prognosis of colorectal cancer.

Many scholars have observed less tumor metastasis in the injured liver, including fatty liver, cirrhosis, and chronic hepatitis B infection [7]. Chronic hepatitis B (CHB) is a serious liver infection and a major health concern worldwide. It is reported that the number of people infected with hepatitis B worldwide is approximately 250 million, and 500,000 people die every year from the complications of this disease [8]. In China, more than 93 million people are carriers of the hepatitis B virus, and about 20 million of them have chronic hepatitis B virus infection [9].

In recent years, some studies have explored the relationship between HBV infection and CRLM [10–19]. Qiu et al. recruited 1298 colorectal cancer patients for a case–control study [13]. They found that HBV infection significantly decreased the risk of liver metastasis [13]. Wang et al. evaluated 344 patients with advanced CRC [12]. Analysis results revealed that the rate of liver metastases was 2.86% and 16.9% in the HBV infected group and the non-infected group, respectively [12]. There were obvious statistical differences between the two groups. Qian and Song et al. presented similar findings [10,11]. However, some other studies showed different opinions. Huo et al. analyzed 4033 colorectal cancer patients and found that chronic HBV infection significantly increased the risk of CRLM [18]. In addition, studies observed that there was no clear relationship between HBV infection and CRLM [17].

So far, the association between HBV infection and CRLM is still unclear. Therefore, we summarize the existing evidences and conduct a meta-analysis to comprehensively assess the risk of CRLM in patients with HBV infection.

## Methods

### Search strategy

A comprehensive literature search was conducted by two independent investigators (RQ.L and WH.K). Relevant studies were systematically searched in the PubMed, EMBASE and Web of Science until 31 October 2020. The following keywords were used: ‘colon cancer’, ‘colon neoplasm’, ‘colon tumor’, ‘colon carcinoma’, ‘rectal cancer’, ‘rectal neoplasm’, ‘rectal tumor’, ‘rectal carcinoma’, ‘colorectal cancer’, ‘colorectal neoplasm, ‘colorectal tumor’, ‘colorectal carcinoma’, ‘Hepatitis B Virus’, ‘HBV’, ‘hepatic metastases’, ‘liver metastases’. Boolean operators, such as ‘OR’ and ‘AND’, were used to combine the above keywords. No language restrictions and manually search for references in the included studies.

### Study selection

The inclusion criteria were as follows: (1) assessed the association between HBV infection and CRLM. (2) offered the numbers of positive/negative liver metastasis persons in the HBV infection groups and control groups, and (3) more than 20 patients with HBV infection were included in the study. The exclusion criteria were as follows: insufficient data studies, animal experiments, letters, case reports and review were excluded.

### Data extraction and quality assessment

Two researchers extracted the data independently (RQ.L and TX.D). The following data were extracted: first author name, publication year, country, study design, age, male/female, the number of patients with liver metastases and non-liver metastases in the HBV group, the number of patients with liver metastases and non-liver metastases in the control group, the number of patients with extrahepatic metastases in the HBV group, and the number of patients with extrahepatic metastases in the control group. All studies were evaluated by the Newcastle-Ottawa Scale (NOS) in meta-analyses [20].

### Statistical analysis

Heterogeneity was assessed based on I^2^. If I^2^ was <50%, a fixed-effect model was used, and if I^2^ was ≥50%, a random-effects model was applied. Odds ratios (OR) and corresponding 95% CI were used to analyze the relationship between HBV infection and CRLM. Meta-regression and subgroup analyses were conducted to explore the sources of heterogeneity. The publication bias was evaluated by Egger’s test. All data analyses were performed using by STATA 12.0 (Stata Corp., College Station, TX, USA). P value <0.05 was considered statistically significant.

## Results

### Brief introduction

The association between Hepatitis B Virus infection and colorectal liver metastasis is still unclear. Therefore, we summarize the existing evidences and conduct a meta-analysis to comprehensively evaluate the association between therefore and colorectal liver metastasis. We firstly searched the designated database to identify the relevant articles and extracted relevant data for meta-analysis. We found that patients with Hepatitis B Virus infection have the reduced risk of colorectal liver metastasis. In addition, HBV infection had no association with extrahepatic metastasis or prognosis of patients with colorectal liver metastasis.

### Search results

**Figure 1.**
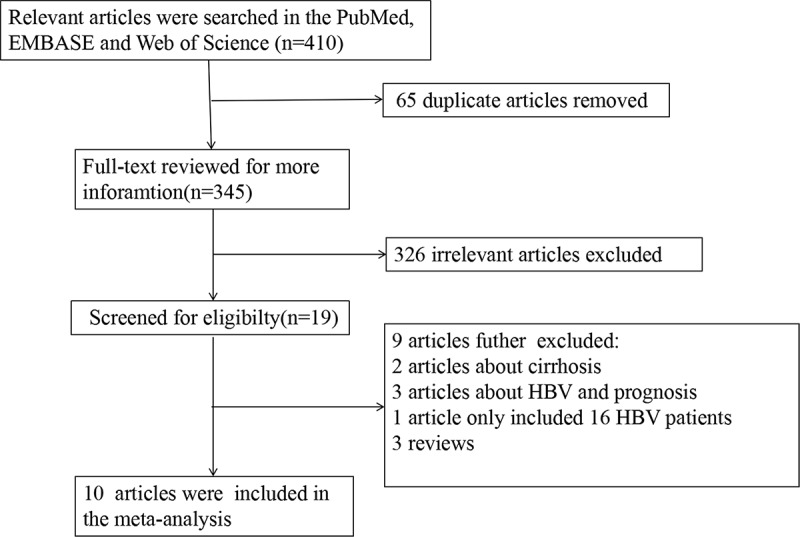
Flow diagram of the literature search

A total of 410 articles were initially collected from specified database. Three hundred and forty-five articles were screened for further information after removing 65 duplicates. Three hundred and twenty-six articles were further excluded after screening the titles and abstracts. Eventually, ten articles which investigated the association between HBV infection and CRLM were recruited in the meta-analysis. The flow diagram of the literature search is presented in Figure 1.

### Study characteristics

The articles included in the meta-analysis comprised two cross-sectional study and eight case–control studies. Two studies were conducted in non-Asian countries, and eight in Asia. There were 17529 participants in the included studies, with the number of participants per study ranging from 356 to 7187. The NOS scores ranged from 5 to 7, with a mean score of 6.4. The characteristics of the included studies are summarized in Table 1.

**Table 1.** Basic information of all included studies

				HBV infection group		Non-HBV infection group				
Study	Year	Country	Study-type	liver metastasis	Non-liver metastasis	liver metastasis	Non-liver metastasis	Age	Male/Female	NOS score
Destri	2012	Italy	Case-control	1	30	43	414	65.7	279/209	6
Qian	2014	China	Case-control	13	125	305	970			7
Song	2001	China	Case-control	10	64	119	319	NA	314/198	7
Utsunomiya	1999	Japan	Case-control	3	34	85	316	NA	245/193	7
Wang	2012	China	Case-control	2	68	48	236	55.5	196/158	6
Qiu	2011	China	Case-control	47	285	272	694	NA	746/552	7
Huo	2018	China	Cross-sectional	38	206	326	3463	NA	2465/1568	7
Qian	2010	China	Case-control	10	104	254	808	59	706/470	6
Iascone	2005	Italy	Case-control	15	72	174	369	65.9	338/292	6
Zhao	2019	China	Cross-sectional	49	315	578	6245	57	NA	5

### HBV infection and CRLM

**Figure 2.**
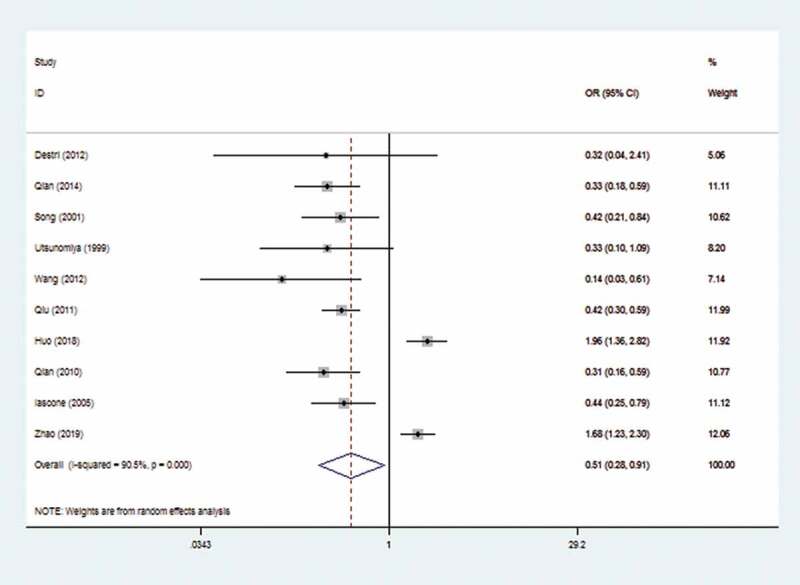
Forest plot of Hepatitis B Virus infection and colorectal liver metastasis

The random-effects model was applied due to the marked heterogeneity (I^2^ = 90.5%). The result showed that there was a significant reduced risk of CRLM in HBV Infection patients (OR: 0.51, 95% CI: 0.28–0.91). The forest plot is displayed in Figure 2.

Subgroup analysis was performed based on the publication year, study type, liver metastases type, sample size, race, case–control ratio, and language (Table 2). The subgroups (cross-sectional study and case–control ratio≥0.5) displayed that HBV infection obviously increased the risk of CRLM (OR: 1.79, 95%CI 1.41–2.27,OR:1.70, 95%CI 1.35–2.15, respectively). In addition, the subgroup of simultaneous showed that HBV infection had no correction with CRLM. The ORs of all other subgroups were consistent with the overall analysis. Regression analysis showed that study type (P < 0.01) and case–control ratio (P < 0.01) are sources of heterogeneity.

**Table 2.** Subgroup analysis and meta-regression of all studies evaluating the association between HBV infection and CRLM

		Pooled OR (95%CI)		Heterogeneity		Meta-regression		
Stratified study	No. of studies	Fixed-Model	Random-Model	I^2^(%)	p-value	Tau^2^	Adj R^2^(%)	p-value
Year						0.3174	42.67%	0.057
>2010	6	0.79(0.66, 0.95)	0.62(0.27,1.38)	93.2%	0.000			
≤2010	4	0.38(0.26, 0.54)	0.38(0.27,0.55)	0%	0.849			
Study type						0.00	100%	<0.01
Cross-sectional	2	1.79(1.41,2.27)	1,79(1.41,2.27)	0%	0.531			
Case-control	8	0.37(0.30,0.46)	0.38(0.30,0.47)	0%	0.870			
liver metastases						0.3704	26.03%	0.175
simultaneous	4	0.88(0.67,1.16)	0.67(0.258,1.744)	89.5%	0.000			
metachronous	3	0.27(0.15,0.48)	0.27(0.15,0.48)	0%	0.180			
Sample size						0.4986	9.94%	0.069
>1000	5	0.77(0.64,0.92)	0.69(0.31,1.56)	94.8%	0.000			
<1000	5	0.36(0.25,0.54)	0.38(0.26,0.57)	0%	0.699			
Country						0.5752	−3.89%	0.328
China	7	0.71(0.60,0.84)	0.55(0.27,1.12)	93.2%	0.000			
Itlay	2	0.43(0.24,0.75)	0.43(0.25,0.76)	0%	0.764			
Japan	1	0.33(0.10,1.09)						
Case-control ratio						0.00	100%	<0.01
≥0.5	3	1.70(1.35,2.15)	1.71(1.21,2.43)	37.6%	0.201			
<0.5	7	0.37(0.30,0.46)	0.38(0.30,0.48)	0%	0.792			

### HBV infection and extrahepatic metastasis

**Figure 3.**
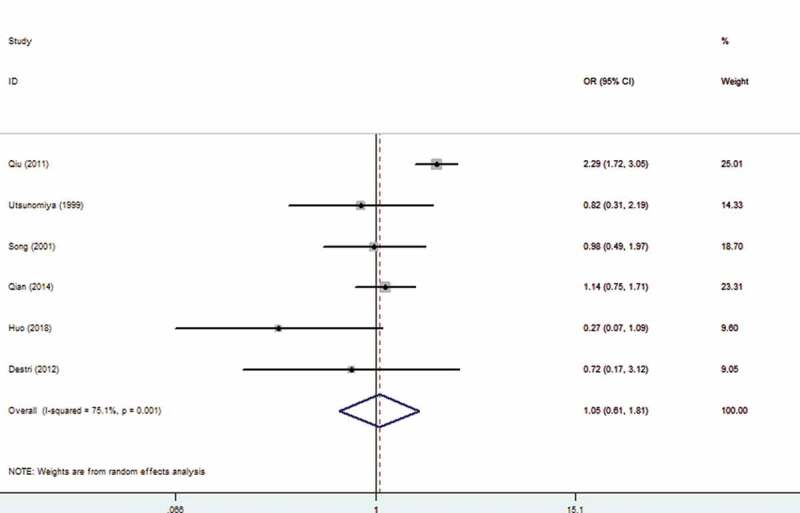
Forest plot of Hepatitis B Virus infection and extrahepatic metastasis

Among the included studies, six studies explored the association between HBV infection and extrahepatic metastasis. The meta-analysis indicated that there was no correlation between HBV infection and extrahepatic metastasis (OR: 1.05, 95% CI: 0.61–1.81) with random-effects model (I^2^ = 72.4%). The forest plot is displayed in Figure 3.

### HBV infection and prognosis of patients with CRLM

**Figure 4.**
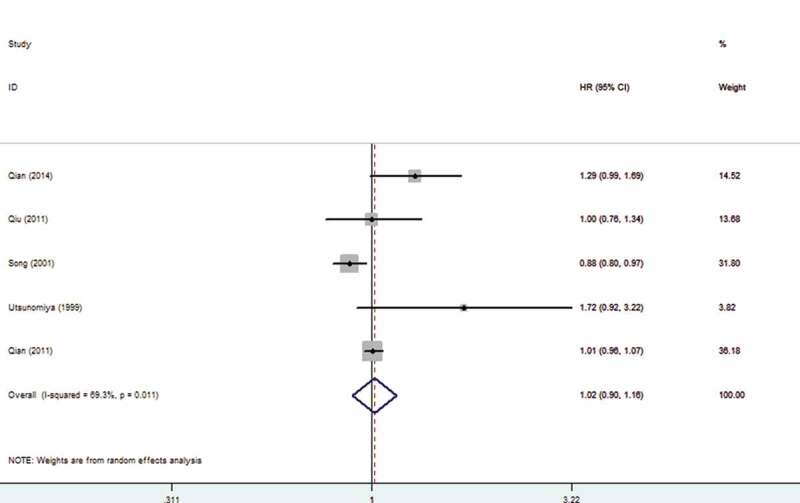
Forest plot of Hepatitis B Virus infection and prognosis of patients with colorectal liver metastasis

We also analyzed the association between HBV infection and prognosis of patients with CRLM. The findings showed that HBV infection did not affect the prognosis of patients with CRLM (OR: 1.02, 95% CI: 0.90–1.16). The forest plot is displayed in Figure 4.

### Sensitivity analysis and publication bias

**Figure 5.**
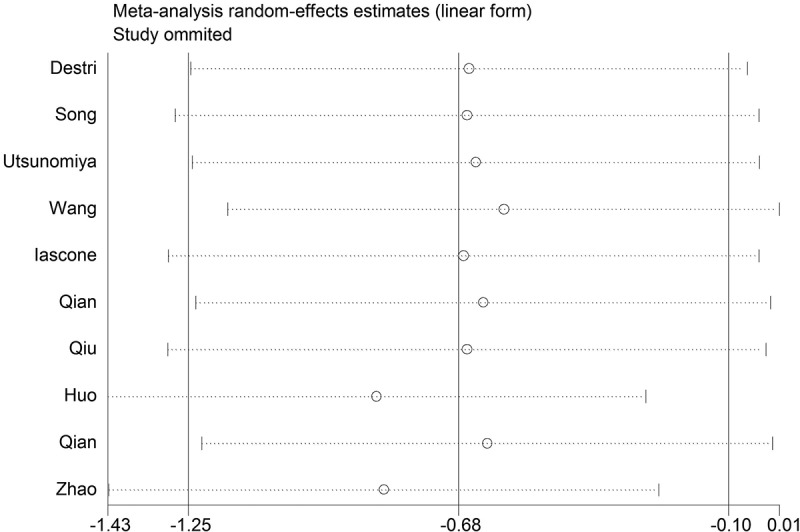
Sensitivity analysis for Hepatitis B Virus infection and colorectal liver metastasis

**Figure 6.**
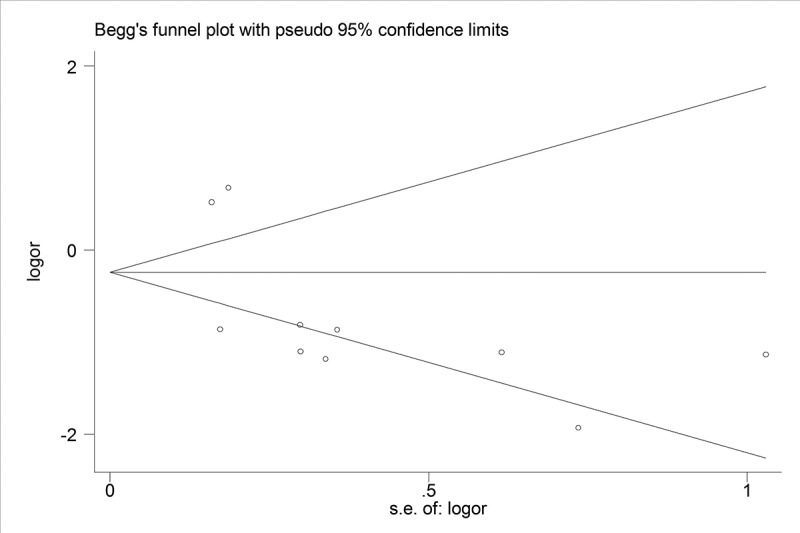
Funnel plots for publication bias for Hepatitis B Virus infection and colorectal liver metastasis

We performed sensitivity analysis to check the stability of the results. The results were consistent with the overall analysis, indicating that the results were stable (Figure 5). Egger’s test was used to p assess publication bias. The P value of Egger’s was 0.092, suggesting that there was no publication bias (Figure 6).

## Discussion

As is well known for us, the study is the first meta-analysis to comprehensively explore the relationship between HBV infection and CRLM. The results indicated that HBV infection was closely correlated with CRLM (OR: 0.51, 95% CI: 0.28–0.91). Subgroup analysis confirmed that HBV infection can reduce the risk of metachronous colorectal liver metastases (OR: 0.27,95 CI: 0.15, 0.48). We also conducted analysis according to different countries, and the results revealed that whether in China or Itlay, HBV infection was significantly related to CRLM. Regression analysis revealed that study type and case–control ratio may be sources of heterogeneity. Zhao et al. suggested that HBV infection was one of the prognostic factors of CRLM [21]. The comprehensive results indicated that HBV did not affect the prognosis of CRLM (OR: 1.02, 95% CI: 0.90–1.16). In addition, HBV infection was not related to extrahepatic metastasis of colorectal cancer (OR: 1.05, 95% CI: 0.61–1.81).

The mechanisms of HBV infection on CRLM are complicated. Lisa firstly discovered that liver cirrhosis was not conducive to the growth of metastatic tumors [22]. Hepatitis B infection leads to liver fibrosis and even cirrhosis. The original structure of the liver is destroyed, and blood vessels are compressed, distorted and even blocked, which reduce the route of tumor metastasis to the liver through blood, thereby reducing the risk of liver metastasis [23]. In cirrhosis, the liver can produce a variety of cytokines, such as TNF-β, IL-1, and platelet-derived growth factor, which inhibit tumor cell metastasis by regulating extracellular matrix [24]. Furthermore, liver cirrhosis can cause a large number of activation of liver Kupffer cells, upregulating the Fas receptor of tumor cells, and promoting Fas-mediated apoptosis of tumor cells [25]. However, Song et al. believed that activation of liver-associated immunity due to HBV replication rather than structural changes during liver damage reduced the incidence of liver metastases in CRC [11]. Lara-Pezzi et al. found that the immunity of patients with HBV infection could be enhanced, thereby increasing the lethality for tumor cells [26]. Hepatitis B virus infection can activate humoral immunity in the liver and promote the production of a variety of cytokines, and they significantly promote the production of extracellular matrix and inhibit the degradation, preventing the adhesion and localization of tumor cells in the live [27]. Additionally, hepatitis B virus also activates the liver’s cellular immunity. On the one hand, a large number of Kupffer cells are activated to directly engulf tumor cells after viral infection [28]. On the other hand, Kupffer cells also promote NK cell activation and induce cytotoxic T lymphocyte generation, further enhancing the killing ability of tumor cells [29,30]. It is also believed that HBV infection affects miRNA expression and mesenchymal–epithelial transformation of tumor cells so as to inhibit colorectal liver metastasis [31,32]. The exact mechanism is not yet fully understood, and further research is still needed.

Several limitations existed in our study. Firstly, the included studies are all retrospective studies, and it is difficult to determine the causal relationship between HBV infection and CRLM. More prospective controlled trials are necessary to validate our findings. Secondly, the results should be treated with caution due to the apparent heterogeneity. Thirdly, most of the included studies are from Asia. The conclusions should be more suitable for Asians, especially for China. Studies on the association of HBV infection with colorectal liver metastasis risk in different regions should be performed. Fourthly, some risk factors that affect colorectal liver metastasis such as diabetes, alcoholism, obesity and smoking cannot be extracted, and the meta-analysis could not be able to exclude their influence. Furthermore, we have not been able to distinguish whether there is a difference in the effects of hepatitis B cirrhosis and chronic hepatitis B on CRLM.

The study also has some advantages. Firstly, our study is the first comprehensive meta-analysis about the effect of HBV infection on CRLM, and the results have higher statistical power and are more persuasive. Secondly, sensitivity analysis revealed that the results were stable. Additionally, we discovered the factors that caused high heterogeneity. More importantly, there is no possible publication bias.

## Conclusion

This meta-analysis confirms that Patients with HBV infection have a reduced risk of CRLM based on current evidence. Undoubtedly, large-scale prospective studies are necessary to validate the present results and seek strategies to prevent CRLM in the future.

## Data Availability

All data are in the manuscript and can be obtained from the corresponding author.

## Abbreviation

HBV, Hepatitis B Virus; CRLM, colorectal liver metastasis; OR, odds ratio; CI, confidence interval; CRC, colorectal cancer; CHB, chronic hepatitis B; NOS, Newcastle-Ottawa Scale.
